# Changes in relationship satisfaction in the transition to parenthood among fathers

**DOI:** 10.1371/journal.pone.0289049

**Published:** 2023-08-30

**Authors:** Judith T. Mack, Lena Brunke, Andreas Staudt, Marie Kopp, Victoria Weise, Susan Garthus-Niegel

**Affiliations:** 1 Institute and Policlinic of Occupational and Social Medicine, Faculty of Medicine, Technische Universität Dresden, Dresden, Germany; 2 Department of Methods in Community Medicine, Institute of Community Medicine University Medicine, Greifswald, Germany; 3 Institute for Systems Medicine (ISM), Faculty of Medicine, Medical School Hamburg, Hamburg, Germany; 4 Department of Childhood and Families, Norwegian Institute of Public Health, Oslo, Norway; University of Rome La Sapienza: Universita degli Studi di Roma La Sapienza, ITALY

## Abstract

To date, research on the transition to parenthood and associated changes in relationship satisfaction (RS) has focused predominantly on mothers with their firstborn. This study targeted fathers to investigate their trajectories of RS with a particular focus on emerging differences between first- and second-time fathers. It furthermore considered various predictors such as the role of age, education, income, duration of relationship, marital status, child’s biological sex, and child temperament. Data from a total of 606 fathers from the prospective longitudinal cohort study DREAM were analyzed. The analyses included assessments of four measurement time points (T1: prepartum; T2–T4: postpartum) over a period of more than 2 years. Latent growth curve modeling was applied with RS as the dependent variable and number of children as one of eight predictors of growth over time. First-time fathers showed higher initial RS, however experienced a steeper decline in the transition to parenthood than second-time fathers. At 8 weeks postpartum, first-time fathers still reported higher RS than second-time fathers. While RS continued to decline for first-time fathers up until 14 months postpartum, second-time fathers experienced an increase in RS. At 14 months and 2 years postpartum, second-time fathers showed higher RS scores than first-time fathers. Similar to first-time mothers, first-time fathers seem to experience a stronger RS decline during the transition to parenthood than second-time fathers, suggesting that especially couples becoming parents for the first time should be prepared for expected changes in their relationship.

## Introduction

Having a satisfying relationship has been identified as one of the most important individual life goals alongside finding real meaning in life [1]. Perceived positive relationship satisfaction (RS) is characterized by a high proportion of shared experiences and high affective involvement of the partners in each other’s life. Specifically for this kind of satisfaction, the transition to parenthood (in the following the process of becoming a parent for the first-, but also the second time, is referred to as transition to parenthood) seems to play a crucial role according to a meta-analysis by Twenge et al. [2]. The importance of this transition is not surprising when taking the following adjustments into account that might lead to less shared experiences and affective involvement: reduction of intimacy or intercourse [2–4], reorganization of the family system [5, 6], stress of adjusting to new roles [7], lack of sleep that often occurs [8], and the additional financial burden of having a child [9]. All these changes may decrease attention paid to the partner and the RS can suffer as a result.

The importance of a good relationship during the transition to parenthood is demonstrated by the study of Figueiredo et al. [10], who showed that the quality of the relationship can have a substantial impact on the parents’ mental health. For examp\le, negative interaction (e.g., many conflicts or low intimacy or support) in the relationship can increase depression symptoms in both mothers and fathers [10]. Relationship discord is among other things also related to poorer outcomes in treatment for mental disorders and even substance use disorders [11]. A decline in RS can also affect participation in child rearing. Fathers with lower RS, for example, show less commitment to parenting [12] and sometimes weaker bonding with their infant [13]. On the other hand, a higher degree of RS can be beneficial for an infant’s personal-social development [14]. In fact, children who grow up in a family with a lot of conflict seem to have a considerably higher probability of developing a mental disorder [15].

Previous research on RS of the past decades has mainly focused on mothers and their first child, whereas fathers as well as subsequent children have received much less attention, as summarized by Redshaw and Martin [16]. A few studies have shown that the birth of the first child is associated with a decline in RS of fathers or couples [6, 17–19], less is known concerning the impact of second or subsequent births [6]. Möller et al. [20] stated that parenthood is stressful for both first- and second-time parents, and if having one child causes stress the second child seems to add even more. In line with this, a few studies found a negative association between RS and the number of children—indicating greater dissatisfaction for couples with more children [2, 21–23]. In contrast, van Scheppingen et al. [8] showed that each additional pregnancy caused less stress compared to the first pregnancy among mothers, probably due to the fact that these mothers already had experience in relation to pregnancy, birth, and caring for the children.

Even though previous studies included couples who had not been first-time parents, they either did not distinguish between first- and second-time parents [17] or used very small samples [24], only mothers [8], or focused mainly on U.S. participants [6]. However, to allow reliable conclusions on this subject, it is important to use larger samples [24] including fathers [8]. In the last decades, fathers have been neglected in research, even though they play as important a role in the family system as mothers. For example, it has been shown that the fathers’ RS was positively associated with father-infant bonding, which is in turn important for the child development [13, 25]. Although a few studies have included couples and thus fathers, as mentioned before, we still know too little about whether or why fathers experience the transition into parenthood and the associated experienced RS differently.

Furthermore, Kluwer et al. [6] calls for a more diverse examination of relationship changes during the transition to parenthood as it is likely that cross-cultural differences exist. This may partly be attributed to great differences of the countries’ particular regulations regarding parental leave or to whether fathers and (especially) mothers are employed at all. In comparison to the U.S., in Germany, e.g. mothers and fathers have the right to take parental leave for a maximum of three years, in which they can receive parental allowance for 12 months. These could even be extended to 14 months if not only mothers but also fathers (or vice versa) take at least two months (so called non-transferable partner months [26]). Such rights and benefits can be important for fathers or families and may account for a difference in results across countries, given paternity leave, for example, was shown to be positively related to parents’ RS [27].

The current study addresses the mentioned gaps with a sample from Germany of fathers with their first or second child. The primary aim of the present study is to examine the potential emerging differences between first- and second-time fathers concerning the trajectories of RS in the transition to parenthood until two years postpartum.

In addition, the secondary aim was to assess the impact of age, education, income, duration of relationship, marital status, child’s biological sex, and child temperament on RS. To date, research has yielded mixed results as to whether or not these variables have a significant influence on the trajectories of RS (during transition to parenthood) or they have rarely been explicitly studied in fathers. Age was found to be either positively correlated with RS in couples [23, 28], negatively correlated for women [22], or not correlated at all [29]. More recent research found RS as a function of age across the life span [30]. Education has been reported to be positively correlated with RS [31, 32], but it is unclear if it can predict RS trajectories during the transition to parenthood. For income however, Doss et al. [17] showed higher income induced a smaller decline in RS during the transition to fatherhood. Contrary to the findings of other studies reporting no effect of income on RS [31, 33]. Duration of relationship was identified as one of the most consistent risk factors for a postpartum decline in RS [17, 34], but meta-analytical findings also indicated small or no effects on RS on the one hand [2, 35] as well as RS as a function of relationship duration on the other [30]. Previous research also suggested relationship dissatisfaction is more typical in cohabiting but not married first-time parents in the peripartum period [36]. In addition, child characteristics like the biological sex and temperament should be considered. The child’s biological sex has been reported to be a risk factor for a postpartum decline in RS of fathers [17] as well as divorce [37], whereas in other studies no effects where found, at least for mothers [8]. Further, having a temperamentally well-regulated infant was significantly related to higher RS [38], although only a small predictive effect of difficult temperament on change in RS was observed [8].

To summarize, the current study directs the focus on fathers and their experiences in the family system with the following research questions: (I) How do first- and second-time fathers’ trajectories of RS develop across the transition to parenthood? (II) Do age, education, income, duration of relationship, marital status, child’s biological sex, or child temperament predict RS during the transition to parenthood?

## Methods

This study is based on data from the ongoing, prospective, longitudinal Dresden Study of Parenting, Work, and Mental Health (**DREAM**; “**DR**esdner Studie zu **E**lternschaft, **A**rbeit und **M**entaler Gesundheit”), which started in 2017. The aim of the study is to investigate the relationship between parental work participation, role distribution, stress factors, and their effects on perinatal outcomes and long-term family mental and somatic health in a community sample. The DREAM study follows a large sample (*N* = 3,860) of expectant mothers and fathers over an extended period of time, providing the possibility to examine pre-, peri-, and postpartum experiences in the transition to parenthood (for further information see [39]). The first measurement (T1) took place during pregnancy (on average 2 months before birth). T2 followed at 8 weeks after the anticipated birth date, T3 at 14 months, T4 at 2 years, T5 at 3 years, and T6 at 4.5 years postpartum.

The DREAM study was reviewed and approved by the Ethics Committee of the Technische Universität Dresden (No: EK 278062015). During recruitment, participants received written information about study aims and procedures. They were informed about pseudonymization and confidentiality as well as the possibility to withdraw from the study at any time. All participants gave written informed consent.

For the current analysis, we report in the following how we determined the sample size, all exclusion criteria, used measures, and we follow the JARS guidelines [40]. Used measures and analysis codes are available at the Open Science Framework and can be accessed at https://osf.io/349vu/?view_only=883c324e7e654e29b59e9da6535d290f. This study’s design and its analysis were not preregistered. Due to data privacy restrictions and ethical restrictions established by the Ethics Committee of the Faculty of the Technische Universität Dresden, the dataset is not publicly available, but only on request.

### Participants and procedure

Recruitment took place in and around Dresden from June 2017 to the end of the year 2020 and was mainly carried out at clinics’ information evenings and birth preparation courses in midwifery practices, as well as through scheduled birth registration by midwives and a freestanding birth center. Apart from this, participants were recruited via bulletins, internet sites, children’s stores, and personal approach. Participants were given the option of taking part either online or with a paper-pencil version sent by postal mail.

**Fig 1:**
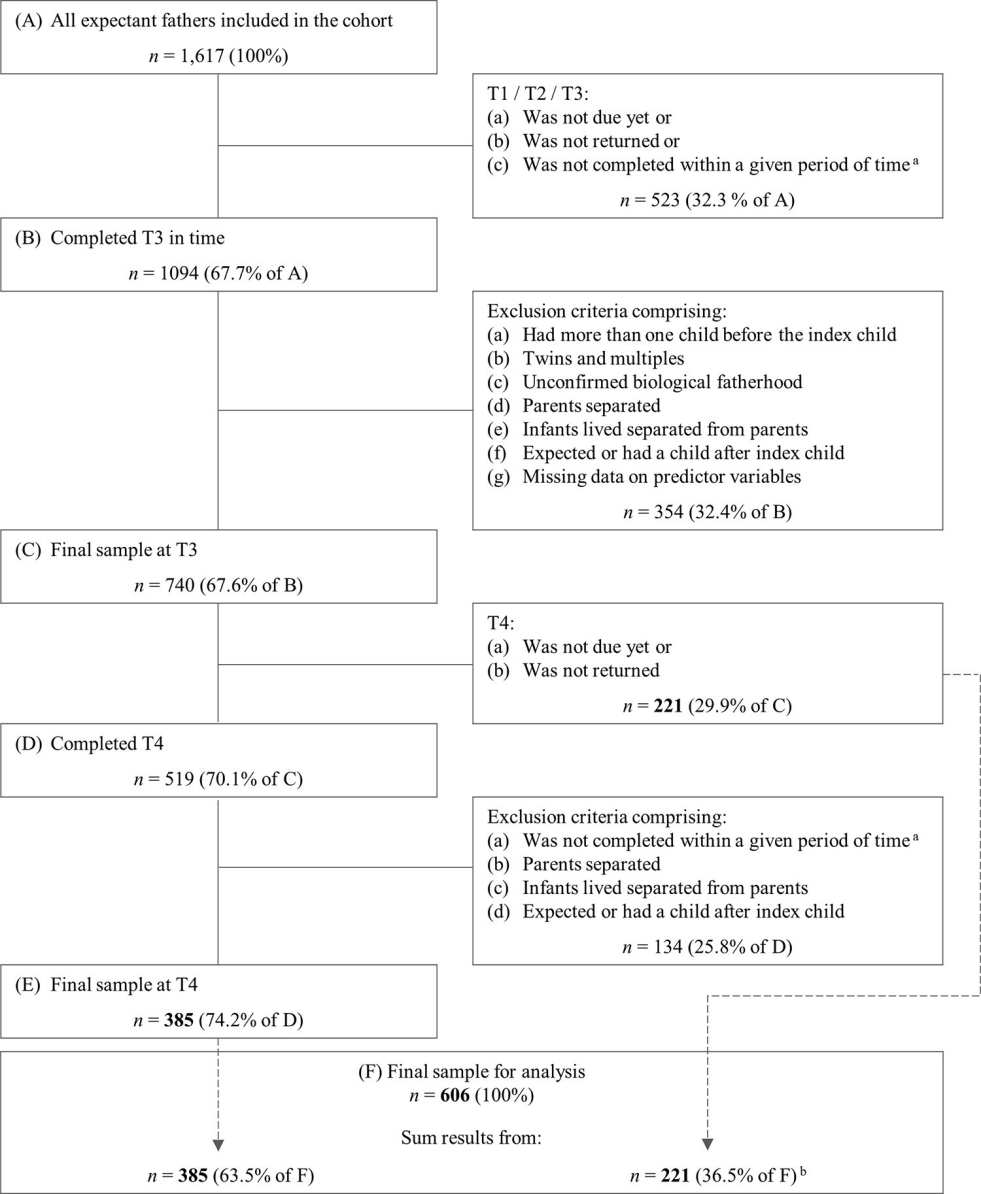
Flowchart of retention rate and exclusion criteria resulting in final sample. T1 = during pregnancy; T2 = around 8 weeks after the anticipated birth; T3 = around 14 months after the actual birth date; T4 = around 2 years after the actual birth date. Numbers in bold represent the final sample. Data from 31^st^ of January 2022 (prospective data collection ongoing). ^a^ Given period of time for T2: within the first 16 weeks after childbirth; T3: within 12 to 16 months after childbirth; T4: within 22 to 26 months after childbirth. ^b^ Participants who participated only until T3 were also included in the final sample, as latent growth curve modeling is tolerant regarding partially missing data (42).

Whereas the DREAM study included 3,860 participants (*n* = 2,243 mothers), the current study focused only on data of the participating fathers (*n* = 1,617), already collected by 31^st^ of January 2022 (prospective data collection ongoing). Data from measurement time points T1 (prepartum) to T4 (2 years postpartum) were considered. The main requirement for being included in the final sample was that fathers provided data on all predictor variables and therefore participated at least until T3 (14 months postpartum). Given that data collection is still ongoing, T3- and T4 were not yet due for all fathers of the 1,617. In addition, it occasionally happened over the four measurement time points that the questionnaires were not always returned. Applying all exclusion criteria (Fig 1), the final sample comprised 606 fathers, with 500 becoming fathers for the first time and another 106 expecting their second child. Exclusion criteria as well as participation and retention rates are depicted in Fig 1.

### Measures

#### Relationship satisfaction

RS was assessed with the short version of the Partnership Questionnaire (PFB-K; [41]). The PFB-K is a modified and shortened version of the Partnerschaftsfragebogen (PFB, 31 items) by Hahlweg [42], which is an established and widely used instrument for measuring RS in German-speaking countries [43]. The original version included three subscales: quarreling, tenderness, and togetherness/communication. A factor analysis showed that all items of the original PFB loaded highly significantly on the general factor RS [44] and correlated highly (*r* = .79, [45]) with another established and more internationally used instrument the Dyadic Adjustment Scale (DAS; [46]). Although these instruments contained different primary factors for measuring RS (e.g., DAS: cohesion, satisfaction, and consensus; PFB: quarreling, tenderness, and communication), the high correlation suggests that an equivalent overall construct of RS can be measured with different areas or factors of relationship quality [44].

For economic reasons, a short version, the PFB-K, was developed and validated by Kliem et al. [41] using a sample of 1,390 participants. The PFB-K contains nine items, such as “We talk to each other for at least half an hour in the evening” and “She gives me a hug”, with response categories ranging from 0 = “never/very rarely” to 3 = “very often”. A sum score is calculated with a maximum value of 27. Higher sum score values indicate a higher degree of couple RS. The total score of the nine items of the short version (PFB-K) has shown to be strongly correlated with the total RS score of the original version of the PFB (*r* = .95; [41]). In the present study, the PFB-K also showed good reliability at T1–T4 (α = .80 –.85). For the interpretation of the PFB-K values, it is stated that an overall raw value of ≤ 13 (maximum value of 27) indicates an unsatisfying relationship [47].

#### Predictor variables

Number of children (i.e., none or one before the birth of index child), age, education, income, duration of relationship, marital status, child’s biological sex, and child temperament were used as predictors. All variables were measured with single items except from child temperament. For education, participants answers were condensed into two categories, (0) ≤ 10 years and (1) more than 10 years of education. Income was measured with the item “What was your average monthly net income in the last 3 months (or a year ago)?” and five response options (1) up to 450€, (2) 451€ to 850€, (3) 851€ to 1.500€, (4) 1.501€ to 2.500€, and (5) more than 2500€. Marital status was dichotomized into (0) not married to current partner (i.e., the child’s mother) and (1) married to current partner.

Child temperament was assessed by the Infant Characteristics Questionnaire (ICQ; [48]). Here, only the fussy/difficult-subscale (our sample: α = .86) of the ICQ was used, which was filled out by the respective father of the child. It contains nine items (e.g. “How does your baby react when you dress him/her?”; “Please estimate the overall difficulty your baby would pose for an average parent.”) with a 7-point Likert scale (1 = not fussy/difficult; 7 = very fussy/difficult), whereby “4” is supposed to represent an average child. For the analyses, the ICQ mean score (range: 1−7) was used.

Of all eight predictors, five (number of children, age, education, income, and marital status) were measured at T1. Child’s biological sex and child temperament were assessed at T2. The duration of relationship (measured with month and year of the beginning of the relationship) was assessed at T3 and is therefore retrospectively calculated for T1.

### Analyses

**Fig 2:**
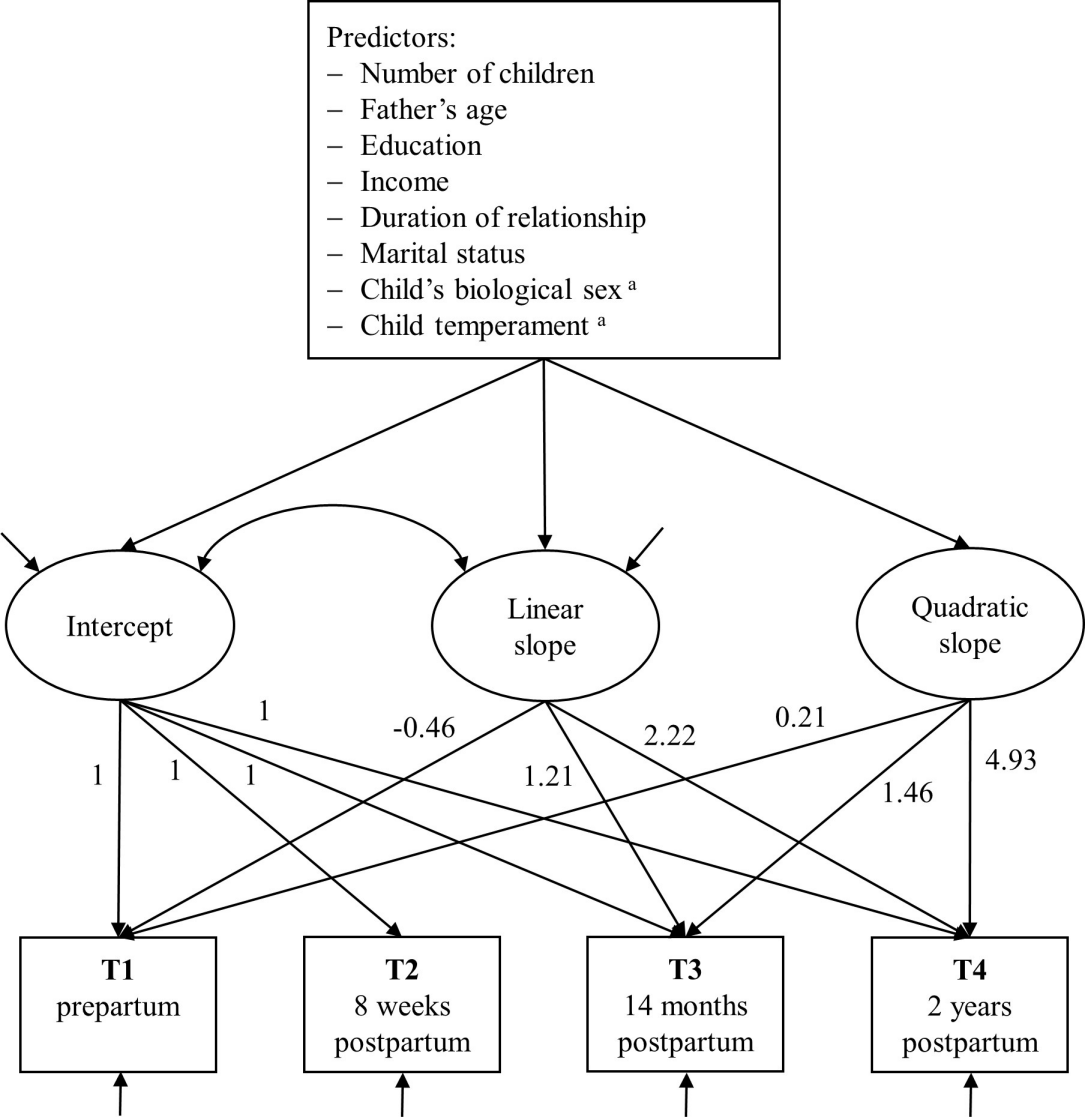
Path diagram of the latent growth curve model. Rectangles represent repeatedly measured relationship satisfaction. Ellipses represent latent growth factors. The variance of the quadratic growth factor had to be fixed to zero to prevent a non-positive latent variable covariance matrix. Arrows represent linear regression relationships. ^a^ Child’s biological sex and child temperament were only used as predictors of the linear and quadratic slope factor, since the intercept represents the level of RS before birth.

To answer the research questions, we applied latent growth curve modeling (LGCM; a multiple regression model), similar to the method used by van Scheppingen et al. [8], Christopher et al. [12], Mortensen et al. [49], and as requested in the meta-analysis by Mitnick et al. [19]. LGCMs offer great flexibility to analyze complex data from longitudinal studies and their trajectories, especially when it comes to developmental and behavioral research [50]. Furthermore, LGCMs are robust regarding varying time periods between measurement points, as it is the case in the DREAM study (months between the different points of measurement: T1−T2: 4.57, T2−T3: 12.17, T3−T4: 10.10). Due to the different time intervals between the measurement points, the following time scores were used: -0.46, 0, 1.21, and 2.22 (unit: 0.1 = 1 month; see Fig 2). Moreover, such LGCMs can handle missing data and longitudinal drop out with a full-information maximum likelihood estimator [51] and allow the addition of predictors [49]. In this study, RS represented the manifest dependent variable. Growth over time was captured by linear and quadratic latent growth factors. An overview of the LGCM can be found in Fig 2.

All analyses were carried out in IBM SPSS Statistics (Version 28 [52]) and Mplus (Version 8.8 [53]). Model comparisons were done using rescaled loglikelihood ratio tests. Model fit was determined using the following fit indices: chi-square test of model fit (χ^2^), the root mean square error of approximation (RMSEA) and its confidence interval, the comparative fit index (CFI), and the Tucker-Lewis index (TLI).

Setting up the LGCM required multiple steps. First, correlations between all predictors were examined to check for multicollinearity. The variable income, which was the only non-dichotomous categorical predictor, was dummy-coded with the lowest category (i.e., below €450) serving as the reference category. Second, unconditional (i.e., without predictors) LGCMs were calculated to find the combination of latent growth factors that represented the development of RS over time best. Third, number of children, age, education, income, duration of relationship (mean-centered), marital status, child’s biological sex, and child temperament (mean-centered) were added as predictors of the latent growth factors. Regressing number of children on the latent growth factors allowed us to calculate RS trajectories for first- and second-time fathers separately, adjusted for all other predictors. Effects were given as unstandardized regression coefficients (*B*) with 95% confidence intervals. Model-implied differences in RS between first- and second-time fathers were given as unstandardized difference scores with standard errors and p-values. All analyses were based on the final sample of 606 fathers (see Fig 1).

## Results

### Sample characteristics

The average age of the fathers was 32.4 years (*SD* = 4.5), covering a range from 20 to 49 years (Table 1). Of the total sample, 75% had more than 10 years of school education. Less than half of the participants were married to their current partner at T1. The average duration of relationship at T1 was 7.3 years (*SD* = 4.1) and 98.5% of all participants lived together with their partner. At the time of completing T1, the female partners were on average in the 30^th^ week of pregnancy. There were significant differences between first-time and second time fathers in age (*t*(604) = −6.039; *p* <. 000), the duration of relationship (*t*(604) = −3.580; *p* < .000), and marital status (*χ^2^* = 6.611; *p* = .010).

**Table 1:** Sample characteristics.

Variable	Total sample	First-time fathers	Second-time fathers
	*n* = 606	*n* = 500	*n* = 106
**Number of children**, *N* (%)			
First	500 (82.5%)	500 (100%)	-
Second	106 (17.5%)	-	106 (100%)
**Age** ^a^, *M* (*SD*)	32.4 (4.5)	31.9 (4.3)	34.7 (4.9)
**German citizenship**, *N* (%)	597 (98.5%)	493 (98.6%)	104 (98.1%)
**German mother tongue**, *N* (%)	592 (97.7%)	487 (97.4%)	105 (99.1%)
**Education**, *N* (%)			
≤ 10 years of schooling	152 (25.1%)	118 (23.6%)	34 (32.1%)
> 10 years of schooling	454 (74.9%)	382 (76.4%)	72 (67.9%)
**Income**, *N* (%)			
Up to 450€	15 (2.5%)	15 (3.0%)	0 (0.0%)
451€ to 850€	6 (1.0%)	5 (1.0%)	1 (0.9%)
851€ to 1,500€	87 (14.4%)	73 (14.6%)	14 (13.2%)
1,501€ to 2,500€	341 (56.3%)	280 (56.0%)	61 (57.5%)
More than 2,500€	157 (25.9%)	127 (25.4%)	30 (28.3%)
**Duration of relationship**^a^, *M* (*SD*)	7.3 (4.1)	7.0 (4.0)	8.6 (4.3)
**Marital status**, *N* (%)			
Married to current partner	269 (44.4%)	210 (42.0%)	59 (55.7%)
Not married to current partner	337 (55.6%)	290 (58.0%)	47 (44.3%)
**Child’s biological sex**, *N* (%)			
Female	292 (48.2%)	238 (47.6%)	54 (50.9%)
Male	314 (51.8%)	262 (52.4%)	52 (49.1%)
**Child temperament** ^b^, *M* (*SD*)	3.2 (0.9)	3.2 (0.8)	3.3 (0.9)

Descriptive Statistics are shown for the total sample and split up for first- and second-time fathers.

^a^Age and duration of relationship in years.

^b^Infant Characteristics Questionnaire, fussy/difficult-subscale; mean score (range: 1−7).

### Relationship satisfaction

**Fig 3:**
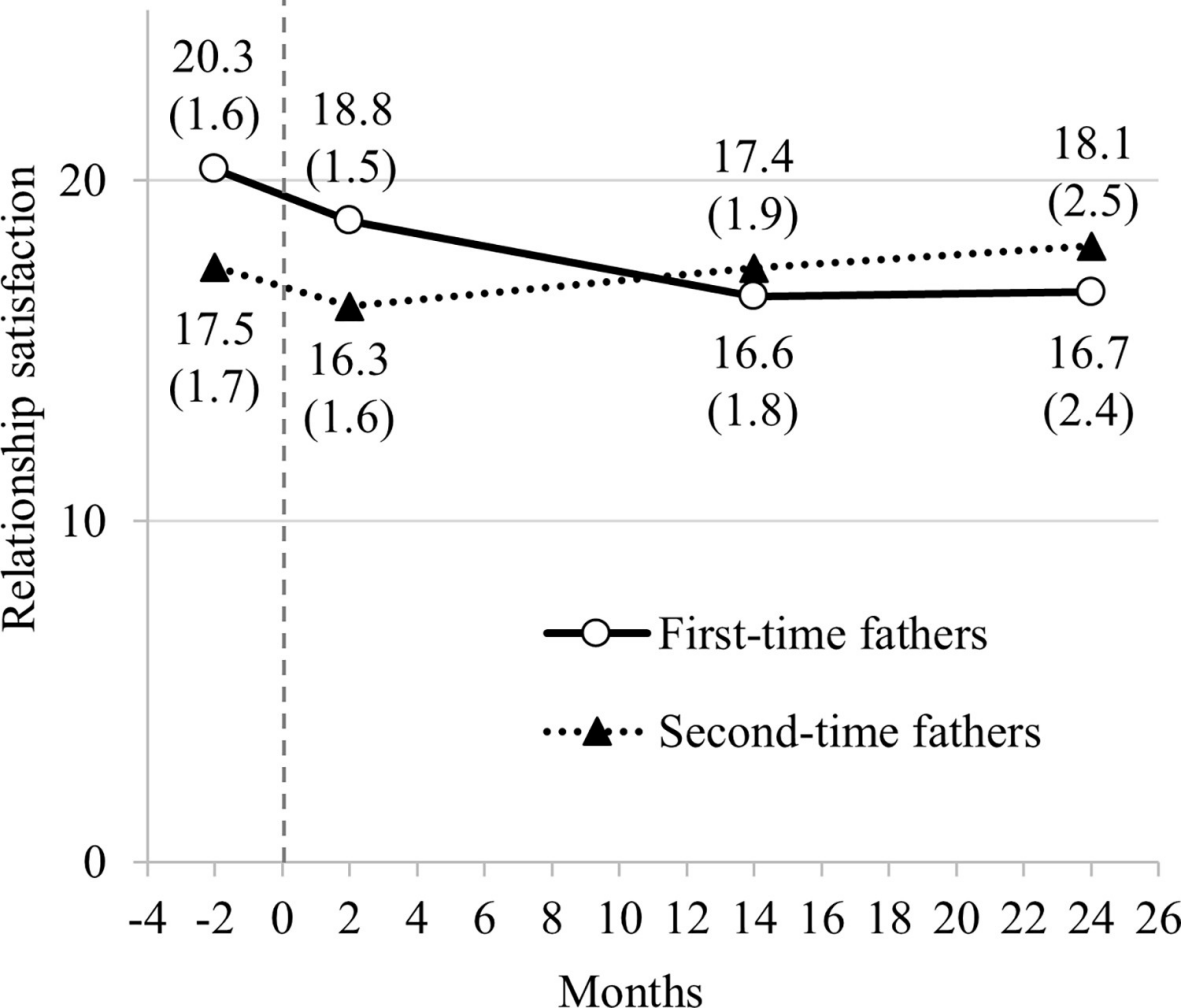
Model-implied trajectories of relationship satisfaction of first- and second-time fathers. Childbirth took place during the period around the dashed grey line. Numbers are estimated means and standard errors in parentheses, adjusted for the fathers’ age, education, income, duration of relationship, marital status, child’s biological sex, and child temperament.

A comparison of the sum score of the total sample with a norm sample of the PFB-K [41] showed significant differences at every measurement point (Table 2). Higher sum score values represent higher level of RS. Furthermore, it was descriptively observed that the percentage of fathers (total sample) who reported a score of 13 or less—indicating an unsatisfying relationship according to Hahlweg et al. [47]—increased from T1 to T3 (T1: 5.9%; T2: 8.3%; T3: 15.7%) and decreased again to T4 (9.9%). A repeated measures ANOVA with the total sample (*n* = 353 completed data for T1–T4) showed significant within-subject differences over time (*F*(2.68, 942.67) = 91.381; *p* < .001; partial *η*^2^ = .206). The Bonferroni post-hoc tests revealed differences between every measurement point (*p* < .001), except between T3 and T4 (*p* = 1.0), which means the RS did not decrease between T3 and T4. The comparison of RS of first- and second-time fathers are presented and described further below with LGCM estimated means and standard deviations (Fig 3).

**Table 2:** Comparison of sum scores of the present sample with the norm sample of the PFB-K.

PFB-K Sum score^a^	Total sample	Norm sample^b^	Group differences
**T1**			
*N*	600	699	*t*(1297) = 8.34
*M (SD)*	20.65 (4.17)	18.71 (4.19)	*p* = .003; *d* = .463
**T2**			
*N*	599	699	*t*(1296) = 3.14
*M (SD)*	19.45 (4.28)	18.71 (4.19)	*p* = .005; *d* = .174
**T3**			
*N*	599	699	*t*(1296) = −2.53
*M (SD)*	18.09 (4.62)	18.71 (4.19)	*p* = .014; *d* = .152
**T4**			
*N*	246	699	*t*(943) = −2.73 *p* = .014; *d* = .178
*M (SD)*	17.84 (4.61)	18.71 (4.19)	

All cells are *M* (*SD*). T1 = during pregnancy; T2 = around 8 weeks after the anticipated birth; T3 = 14 months postpartum; T4 = 2 years postpartum. All values of the present total sample differed significantly from the norm sample at every time point (*p*-values were adjusted via Bonferroni-Holm correction).

^a^ Sum score of the nine PFB-K items; values range from 0–27.

^b^ Men norm sample; age range: 19–89 years.

### Intercorrelations

Five of the correlations between the predictors were statistically significant, but none were greater than *r* = .36 (see Table 3). Based on the correlations in Table 3, it could be assumed that multicollinearity was not a problem in the planned regression analysis.

**Table 3:** Correlations of predictor variables included in the latent growth curve model.

Predictors	1.	2.	3.	4.	5.	6.	7.	8.
1. Number of children	**—**							
2. Age	.24***	**—**						
3. Education	−.07	.00	**—**					
4. Income	.05	.31**	.12**	**—**				
5. Duration of relationship	.14**	.15**	.07	.10*	**—**			
6. Marital status	.10*	.02	.03	.08	.36**	**—**		
7. Child’s biological sex	−.03	.03	−.09*	−.04	−.01	−.07	**—**	
8. Child temperament	.02	−.02	.06	.04	.03	.05	.06	**—**

*p < .05

**p < .01

***p < .001.

### Latent growth curve model

The LGCM with linear and quadratic growth factor fit the data best. As the initial quadratic model resulted in a non-positive latent variable covariance matrix (psi), the variance of the quadratic growth factor was fixed to zero. The final model including all predictors provided excellent fit to the data (χ^2^(17) = 21.67, *p* = .20, RMSEA = 0.021, 90% CI [0.000, 0.045], CFI = 0.996, TLI = 0.989) and yielded the following parameter estimates for the latent growth factors describing first-time fathers’ relationship satisfaction over time at the average of all covariates: Intercept = 18.82 (95% CI [15.90; 21.74]), Linear Slope = -2.90 (95% CI [-5.80; -0.01]), and Quadratic Slope = 0.88 (95% CI [-0.66; 2.43]).

First-time fathers showed higher initial RS (Diff_pregnancy_ = 2.88, SE = 0.46, *p* < .001) but experienced a steeper decline in the transition to parenthood than second-time fathers (Fig 3 and Table 4). At 8 weeks postpartum, first-time fathers still reported higher RS than second-time fathers (Diff_8 weeks pp_ = 2.51, SE = 0.44, *p* < .001). While RS continued to decline for first-time fathers up until 14 months postpartum, second-time fathers experienced an increase in RS. At 14 months (Diff_14 months pp_ = -0.83, SE = 0.21, *p* < .001) and 2 years postpartum (Diff_14 months pp_ = -1.34, SE = 0.44, *p* = .002), first-time fathers showed lower RS scores than second-time fathers. Linear regression results predicting the latent growth factors are shown in Table 4.

**Table 4:** Linear regression results predicting the latent growth factors of relationship satisfaction.

	Dependent variables (relationship satisfaction)					
	Intercept		Linear slope		Quadratic slope	
	*B*	*95% CI*	*B*	*95% CI*	*B*	*95% CI*
Number of children (*Ref*.: First-time fathers)	**-2.51**	**[-3.38; -1.64]**	**0.78**	**[0.16; 1.41]**	-0.08	[-0.42; 0.26]
Age (in years)	0.05	[-0.02; 0.13]	0.02	[-0.04; 0.08]	-0.02	[-0.05; 0.02]
Education (*Ref*.: ≤ 10 years of school education)	0.30	[-0.45; 1.04]	0.06	[-0.54; 0.67]	0.07	[-0.24; 0.38]
Income (*Ref*.: ≤ 450€)						
451–850€	0.14	[-3.17; 3.45]	0.11	[-2.97; 3.19]	-0.04	[-1.50; 1.45]
851–1,500€	-0.70	[-2.65; 1.24]	0.35	[-1.83; 2.52]	0.06	[-1.21; 1.31]
1,501–2,500€	-0.83	[-2.60; 0.94]	0.13	[-1.99; 2.26]	0.14	[-1.11; 1.38]
> 2,500€	-1.07	[-2.91; 0.77]	-0.19	[-2.38; 2.00]	0.34	[-0.93; 1.61]
Duration of relationship at T1 (in years)	**-0.14**	**[-0.23; -0.06]**	0.01	[-0.06; 0.08]	0.01	[-0.03; 0.04]
Marital status (*Ref*.: Not married)	0.16	[-0.52; 0.84]	-0.20	[-0.73; 0.34]	0.12	[-0.16; 0.40]
Child’s biological sex (*Ref*.: Female)	-	-	0.07	[-0.44; 0.59]	-0.04	[-0.31; 0.23]
Child temperament	-	-	-0.22	[-0.32; 0.28]	0.00	[-0.16; 0.17]

Cells are unstandardized regression coefficients (*B*) and its 95% confidence intervals. Numbers in bold represent statistically significant results (*p* < .05). T1 = during pregnancy. Duration of relationship and child temperament (ICQ mean score) were mean-centered. The child’s biological sex and child temperament were only used as predictors of the linear and quadratic slope factor since the intercept represents the level of relationship satisfaction before birth.

## Discussion

This study aimed to investigate fathers’ trajectories of RS across the transition to parenthood, with a particular focus on emerging differences between first- and second-time fathers. Altogether, it was revealed that the birth of a child—regardless of whether it is the first or second—is associated with a decline in RS among fathers. However, differences became apparent regarding the number of children, which was identified to have the greatest association with RS concerning initial value and temporal course. First-time fathers showed a higher level of RS before birth than second-time fathers and a steeper decline after birth. The groups differed in their RS at each measurement point. At 8 weeks postpartum, first-time fathers still reported higher RS than second-time fathers. While RS continued to decline for first-time fathers up until 14 months postpartum, second-time fathers seemed to experience an increase in RS. At 14 months and 2 years postpartum, second-time fathers showed higher RS scores than first-time fathers and seemed to be at their baseline level during pregnancy. Apart from this, the duration of relationship showed a significant association with the initial values of RS. Fathers in longer-lasting relationships exhibited lower RS before birth. In this study, age, education, income, marital status, the child’s biological sex, and temperament did not predict the RS in the transition to parenthood.

### Primary research question

The results of our study concerning the number of children (i.e. number of children associated with RS, higher RS during pregnancy for first-time fathers, and decline after birth for both groups) are consistent with previous literature [2, 19, 21–23]. It is also largely in agreement with the results found among mothers by van Scheppingen et al. [8], except that they could not depict an increase in RS for second-time mothers after the RS decline after birth.

Nonetheless, our results are partially inconsistent with the study by Figueiredo and Conde [24]. These are, to the best of our knowledge, currently the only study that has also examined the differences in RS trajectories of fathers with their first child compared to fathers with their second child. Analogous to the present study, the authors reported that second-time parents had significantly lower scores in RS than first-time parents during pregnancy. However, the authors found no differences between these two groups postpartum (conducted up to 18 months after birth), whereas in the present study differences in the trajectories of RS appeared to be significant even two years after birth and at every time point, RS differed between first- and second-time fathers. These different findings might be related to the comparatively small sample of Figueiredo and Conde (n = 21 first-time fathers, n = 20 second-time fathers), the potentially insufficient statistical power [24], the different statistical approach, and different a measure for RS. Another explanation could be possible cultural (role of fathers, family systems) or legal differences between Portugal and Germany, which can result in different benefits, difficulties, and resources for parents, which in turn can have an influence on RS and therefore, result in slightly different trajectories. There are, e.g., some recent studies that reveal a connection between parental leave and parental RS [27, 54–56], indicating parental leave taking is positively associated with relationship quality and RS of mothers and fathers. In addition, interestingly, RS of mothers and fathers decreases with increasing length of solo maternal leave [54]. Parental leave options in Germany (12 months paid parental leave, and 2 months bonus leave if both parents take at least two months of leave) offer a longer paid duration and more incentives for the partner to take leave, and thus probably more incentives, flexibility, and security for fathers than in Portugal (6 months paid parental leave and one month of bonus leave if both parents use parental leave). This could also be especially important for families of second-time fathers, assuming that more children mean more resources and time needed for childcare. However, to actually support these possible reasons for the differences in our samples, data on paternity leave take-up and duration would be needed. Apart from that, Figueiredo and Conde [24] only cover a period up to 18 months postpartum, where the trajectories of their first- and second-time fathers began to converge. It is not known whether their trajectories continue to change shortly after 18 months, resulting in an increase of RS of second-time fathers and rising above that of first-time fathers, which would be consistent with the trajectories in our sample only slightly (4–5 months) later.

The finding of an increase of RS for second-time fathers from 14 months postpartum is novel and has also not been reported among second-time mothers [8]. Nevertheless, our outcome could be a first indication for the rebound of RS after a certain period of time [5, 57]. Likewise, studies have shown that RS can increase again, with findings indicating that RS levels are greatest when children are between 8 and 12 years of age [57]. Assuming that as children become more autonomous, less time is invested in childcare. Consequently, more time could be available for partnership care, which for second-time fathers could already begin after the first 14 months. First-time fathers did not show such a rebound in the first two years postpartum, suggesting that first-time fathers and their relationships need more time to adjust to the changes brought about by the new family member than second-time fathers, who are likely to benefit from their experiences. Overall, our results show that having a child seems to negatively affect fathers’ RS—both immediately after birth and within the next two years, however, with second-time fathers’ RS recovering and returning to baseline during these two years after birth.

Apart from this, the comparison between the present study sample and the norm sample [41] revealed significantly higher values of our sample on the sum score of the PFB-K at the time point of pregnancy and eight weeks postpartum. It should be noted that of the norm sample, only 6.6% reported having a child under the age of 3 years. One possible reason for the higher values at the beginning of the study might be that the couples were pregnant during T1 and therefore, in accordance to Huss and Pollmann-Schult [5], might have experienced a peak in RS due to pregnancy, which was followed by a decline after birth. Lawrence et al. [18] refer to this idea as the transition to pregnancy effect, which is to describe the idea that RS increases from pre-pregnancy to pregnancy. Hence, if couples are surveyed for the first-time during pregnancy, no statement can be made about the baseline level of RS before the time of pregnancy and as a result the effect of becoming a parent might be overestimated [17]. That means the decline after birth for first-time parents could also be just a decline of the transition to pregnancy effect [5]. However, this would not explain the decline after birth and the following increase during two years postpartum for second-time fathers, even considering that the transition to pregnancy effect is smaller or absent for subsequent children. This suggests that there may nonetheless be a decline in fathers’ RS due to birth and associated changes (and not just adjustment to pre-pregnancy levels). The magnitude of the decline could depend on the magnitude of the increase due to the transition from pre-pregnancy to pregnancy (i.e., steeper decline for first-time fathers). Still, this can only be confirmed if future research measures RS before pregnancy and at least two years postpartum.

### Secondary research question

The secondary aim of this study was to identify predictors of fathers’ RS, namely, to assess the impact of age, education, income, duration of relationship, marital status, child’s biological sex, and child temperament on RS of fathers during the transition to parenthood.

#### Age, duration of relationship, and marital status

In our analyses age showed a tendency toward significance, in the direction that older fathers showed higher RS before birth—which would be in line with previous research of couples [23, 28]. The significant effect of duration of relationship, on the other hand, suggested that longer relationship duration is associated with lower RS before birth. Although, it was not significantly related with the change over time. In this context, it is difficult to draw a comparison with previous studies, as these usually examined the trajectories over time and do not report the impact of the predictors on the initial values separately. Nevertheless, many studies reported a positive association of a longer duration of relationship on the RS [17, 19, 34]. Reasons for the varying results could be, on the one hand, different methods and measures. On the other hand, our sample seems to be older and reports a longer relationship duration. Focusing on a larger time frame than the transition to parenthood, a recent meta-analysis shows that RS decreases and increases across the life span [30]. It showed having children predicted lower mean levels of RS and, independent of children, the trajectories of RS differ between a function of age and a function of relationship duration. RS as a function of age across the life span appeared U-shaped with a decrease to a low point at 40 years of age and an increase thereafter. This again contrasts partially with the findings that older men (i.e., fathers, at least until the age of approx. 40 years) reported higher RS during pregnancy, although not in the light of trajectories. For relationship duration, the RS trajectories were more complex and dynamic. With respect to the period in which our sample falls, Bühler et al [30] found that RS decreased within the first 10 years of relationship and reached a low point at 10 years. This supports our findings that overall RS decreases with increasing relationship duration, at least up to a certain point in the course.

Marital status during pregnancy could not predict the initial or course of RS in our sample, although some previous evidence stated that relationship dissatisfaction is more typical in cohabiting but not married first-time parents in the peripartum period [36]. Nevertheless, our results are in line with the meta-analysis of Bühler et al. [30], which showed that being married has no predictive value for relationship satisfaction across the life span. It is important to note that we cannot exclude the possibility that unmarried fathers at T1 married within T2–T4, as this was only assessed during pregnancy.

In addition, in our sample second-time fathers were older, more often married, and had a longer duration of relationship than first-time fathers. Typically, these characteristics as well as the number of children are positively related but may play different roles for RS at different stages of life.

#### Education and income

Interestingly, neither education nor income appeared to be significantly associated with the initial values of RS or the corresponding trajectory of the fathers, which is in contrast to some previous literature based on samples of couples (education: [21, 31, 32]; income: [17, 58]). However, a few previous studies also reported no correlation of income and RS [31–33, 35]. It is worth mentioning again, that different results may emerge in a multivariate model when adjusting for a different set of predictors (as it was the case with the listed studies) or simply because education was operationalized differently. Thus, a possible reason why income alone did not turn out to have a significant influence on RS might be the fact that it is only one aspect to consider when it comes to the career. Factors like fear for one’s own workplace, fulfilment with one’s profession, or work-family conflict might be highly important for the RS as well [17, 59].

#### Child’s biological se

Regarding the child’s biological sex, our study showed—similar to van Scheppingen et al. [8]—no significant correlation and contradicts numerous studies, based on U.S. samples, that showed positive effects for male offspring [17, 37, 60]. Our results indicate that, at least among our German sample, RS does not depend on the biological sex of the child. Accordingly, cross-cultural differences could be the cause of the differing results [37].

#### Child temperament

Child temperament was considered to have an impact on the trajectory of RS most likely, as no published study with contrary evidence was found. Our results—no significant association of child temperament and fathers’ RS—are therefore not in line with previous reports [38, 61], whereas van Scheppingen et al. [8] also only reported a small effect of difficult temperament on change in RS. For the explanation of our results, the age of the children, whose temperament was rated, could be decisive. The children in the present study were as young as 8 weeks at the time of assessment, whereas all other reported studies included children with an average age of 6 months. However, other authors successfully used the ICQ on children as young as ours or even younger [62–65]. Thus, it can be assumed that the ICQ is appropriate for assessing temperament, but it may take time before the association of the child temperament and parents’ RS becomes apparent. Besides this, children’s temperament could influence parent’s RS only indirectly, i.e. a more difficult temperament leads to more parental demands, energy, and stress [66, 67], which in turn could lead to less time and satisfaction in the relationship [35, 68]. This is suggested by the spill-over theory, which states that family subsystems (e.g. parental subsystem and marital subsystem) are interdependent and (e.g.) stress from one subsystem will be transferred to another [69]. However, apart from the general spill-over effect in family subsystems, evidence on relations within temperament, stress, and RS is not fully consistent, mostly focused on mothers, and suggested gender differences. At least our results show no direct association between child temperament and fathers’ RS within two years postpartum.

### Implications

Being aware of a possible decline of RS after birth and also a possible rebound in the long run could contribute to less suffering in the first year postpartum. This knowledge should be addressed as psychoeducation in antenatal classes to help couples prepare and understand that a decline in RS might be a “common” process of adjustment.

Furthermore, in the very best case, these results would also help to systematically predict, which fathers are more likely to experience a stronger decline of RS during transition to parenthood. Especially the differentiation between first- and second-time fathers might be of great use. However, as most of the other predictors in our study did not show an association, more research is required regarding this matter. Such insights could subsequently be incorporated to provide additional supportive measures and prepare those couples for the expected strain on the relationship, which might prevent unnecessary stress, potentially mental health issues, or even separation or divorce.

Apart from the complexity of predicting which couples are likely to experience a decline in RS, it is worth to briefly repeat the potential causes for this decline. Many studies mention the reduction of intimacy or intercourse [2, 3], whereas others report the general reorganization of the family system as a potential cause [5, 6]. In this regard, it shall be emphasized that mothers and fathers also provide different reasons in some cases. Fathers, for example, were shown to increasingly mention the financial pressure that is associated with becoming a parent [9]. Generally, it can be argued that the birth of a child often leads to a shift in focus, which results in less attention being paid to the partner. Consequently, partners may feel less close and supported by each other [70]. Furthermore, previous research has shown that a lot of conflicts, little intimacy, or low RS can lead to psychological disorders [25] and poorer father-infant bonding [13], which in itself shows the importance of a well-functioning and healthy partnership [24].

Nevertheless, it is important to keep in mind that RS alone does not determine a person’s overall life satisfaction—instead, it only represents one aspect of it. It might therefore be possible that, although RS declines during the transition to parenthood, the feeling of having found a meaning in life increases through the birth and care of one’s own child [2]. This feeling of having found a meaning in life (categorized under personal growth goal) is considered as one of the greatest goals in life besides a satisfying relationship (categorized under relationship goal; [1]).

### Strengths and limitations

To the best of our knowledge, the present study is the first to shed light on the trajectories of RS and the according differences between first- and second-time fathers over the transition to parenthood until two years postpartum. Noteworthy strengths of the DREAM study are the comparatively large sample size, the use of a standardized questionnaire to assess RS (instead of using just a single item), the long period of time covered, and its multiple measurement points [18]. It is furthermore unique that not only parents with their first child were included, as it was the predominant case during the last decades [7, 17, 19]. Alongside the studies from the U.S., Norway, and Portugal, our sample is also the first from Germany addressing the aforementioned questions, which is of great relevance due to possible cross-cultural and political differences. Whereas large studies to date often focused mainly on mothers, the present study focuses specifically on fathers. Thus, our study complements an area of research that has been rarely examined and adds new information to the limited knowledge on the RS of fathers’ and related predictors.

Although the present study provides new insights with many strengths, the following limitations must be acknowledged. First, the used sample might suffer from selection bias. Most of the analyzed participants had a higher level of education (56.8% of our final sample holds a university degree) than the general German male population (33.9%; [71]). Moreover, expectant parents were recruited at clinics’ information evenings or birth preparation courses, which may explain the larger number of couples expecting their first child. Experienced parents expecting their second child might be less interested in such events, resulting in the significantly smaller group of second-time fathers (*n* = 106 vs. *n* = 500). In addition, it can be assumed that couples with a more intact RS are more likely to participate in these events, as well as in our study about family systems and the transition to parenthood in the first place and over the long term. These biases limit the generalizability of the findings.

Second, it cannot be ruled out that the same decline would have also occurred without the birth of the child. Accordingly, it is not self-evident that the birth of the child is the causal reason for the decline in RS [17, 19]. This issue was however addressed by a meta-analysis by Twenge et al. [2], which found a significant effect across 90 studies (all of which included expectant parents and nonparents) that nonparents showed a higher average RS than parents [18, 57], which was confirmed later in another meta-analysis [30]. Similarly, the authors showed that the transition to parenthood was associated with a greater decline in RS, which invalidates above mentioned limitations to a certain extent. For a clear evaluation of this issue, it would however be reasonable to include a control group with couples not expecting a child, which would provide a more reliable attribution of the decline in RS to the transition to parenthood [7, 19].

Finally, data assessment took partially place during the COVID-19 pandemic. This means that the pandemic and the resulting restriction measures affected included fathers either not at all or at a different time between T1–T4, which could have had an impact on their RS. In the current dataset, there were not yet enough assessed data for the necessary multi-group model, since fathers whose child was born during the pandemic had not yet completed T4. This is why we could not look into the impact of COVID-19 pandemic on RS over the course of two years postpartum.

### Future research

Future research should continue to investigate the transition to parenthood for fathers with predictive, risk as well as protective factors for RS. It should further examine comparisons between mothers and fathers and their respective development of RS in the transition to parenthood using dyadic designs (similarly to Figueiredo and Conde [24] but with a larger sample) to see what role specific actor and partner effects play. Research should also make an explicit distinction between subsequent children because our research showed clear differences due to number of children. To rule out the transition to pregnancy effect (increased RS due to pregnancy [18]), longitudinal studies assessing RS before pregnancies could make an important contribution to this research. Furthermore, it would be of great value to examine the found results over a longer period than 2 years postpartum, given the differences in RS at the end of the observed time period. In addition, it would also be interesting to see what effects subsequent pregnancies have or how changes in relationship satisfaction affect the likelihood of having future children.

Moreover, to gain further understanding of which couples are at particular risk of showing a severe RS decline during the transition to parenthood, the set of predictors should be expanded or varied (e.g., mental health, planned/unplanned pregnancy, working conditions, handling of childcare and childrearing, or the sleeping behavior of the child).

## Conclusion

In summary, the birth of a child seems to be generally associated with a decline in RS of fathers, regardless of whether it is the first or second. Nevertheless, differences were evident: first-time fathers showed a higher level of RS before birth than second-time fathers and a steeper decline after birth. Second-time fathers’ RS bounced back within the two years postpartum period, indicating that the observed decline in RS might only be temporary, and the adaptation process could take more time for first-time fathers. With evolving autonomy of the children and increasing parenting experience, fewer resources must be invested in childcare, which in turn opens time for taking care of the partnership.
